# Editorial: Climate impact on plant holobiont: mitigation strategies and sustainability, volume II

**DOI:** 10.3389/fmicb.2024.1503816

**Published:** 2024-10-21

**Authors:** Shekhar Jain, Anukool Vaishnav, Devendra Kumar Choudhary

**Affiliations:** ^1^Faculty of Life Sciences, Mandsaur University, Mandsaur, Madhya Pradesh, India; ^2^Department of Biotechnology, GLA University, Mathura, Uttar Pradesh, India; ^3^Amity Institute of Microbial Technology, Amity University, Noida, India

**Keywords:** climate change, plant holobiont, bio-control, soil microbes, sustainable agriculture

## Introduction

As the global climate crisis accelerates, it becomes increasingly clear that the future of our planet's ecosystems hinges on understanding complex biological interactions (Fletcher et al., 2024). The plant holobiont, comprising the host plant and its associated microbial communities, is crucial for plant health, growth, and resilience. However, climate change is disrupting this delicate relationship, influencing temperature, precipitation patterns, and the occurrence of extreme weather events (Noman et al., 2021). These environmental shifts can disturb plant-microbe interactions, leading to both biotic and abiotic stress, reduced agricultural productivity, and biodiversity loss. Climate-induced changes in soil moisture and nutrient availability can also alter the composition and functioning of the plant microbiome, often diminishing the plant's capacity to withstand stressors like drought, diseases, and pests (Vaishnav et al., 2023).

Mitigation strategies that focus on the plant holobiont are vital for addressing climate change challenges and promoting sustainability. Key measures include utilizing beneficial microbes like plant growth-promoting rhizobacteria (PGPR) and mycorrhizal fungi to enhance plant stress tolerance (Jain et al., 2018). Sustainable farming practices such as crop rotation, organic farming, and reduced chemical inputs further support healthy plant-microbe relationships by preserving soil biodiversity (Choudhary et al., 2016). Innovations in microbial inoculants, precision agriculture, and genetic engineering also offer promising avenues for optimizing holobiont resilience under climate stress, ensuring both agricultural and ecosystem sustainability (Marco et al., 2022).

The first volume of *Climate impact on plant holobiont* presented 16 papers, including reviews and original research (Choudhary et al., 2023). The second volume, *Climate impact on plant holobiont: mitigation strategies and sustainability*, continues this crucial discussion. It explores plant-microbe relationships under climate stress and provides actionable strategies for promoting sustainability in agriculture and ecosystems. Comprising six research articles, this volume delves into the inter-relationship between plants, microbes, and climate change, offering insights for developing mitigation strategies.

## Climate impact

Greenhouse gas emissions, particularly nitrous oxide (N_2_O), are significant drivers of global climate change. Nitrogen, a key element for all living organisms, plays a vital role in plant nutrition by enhancing crop biomass and grain yields (Jain et al., 2021). However, nitrogen fertilizers also alter soil microbial communities, particularly denitrifying bacteria, which can increase N_2_O emissions, a potent greenhouse gas that contributes to global warming and ozone depletion. A recent study examined the relationships between nitrogen fertilization, soil denitrification, N_2_O emissions, potential denitrification activity, and maize nitrogen use efficiency (NUE) in semi-arid regions. The findings revealed that while higher nitrogen fertilization improved maize productivity and NUE, it also elevated N_2_O emissions by modifying the composition and interactions of denitrifying microbes containing the *nirS* and *nosZ* genes in soils. Therefore, the fertilizer optimization is crucial for the plant growth and climate sustainability. The study concluded that 200 kg N ha−1 yr−1 is optimal for balancing increased maize yield with reduced N_2_O emissions in China's semi-arid Loess Plateau (Fudjoe et al.).

Ecosystem parameters like soil, microbial communities, and climate are interconnected, and understanding their relationship is key to sustainable management and conservation (Aqeel et al., 2023). The research study on seasonal climate fluctuations shows that soil properties and microbial populations vary with seasons, significantly influencing soil conditions and microbial activity. During summer, soil pH, moisture, electrical conductivity, and organic carbon content were higher than in winter, primarily due to shifts in microbial activity (Solanki et al.). These insights are essential for biodiversity conservation and ecosystem management, particularly in tropical dry deciduous forests.

## Plant-microbe interaction

Genome sequencing of pathogens is crucial for understanding pathogenicity genes and host-pathogen interactions (Gurjar et al., 2022). In a study on the whole genome sequencing of *Tilletia caries*, the pathogen responsible for common bunt in wheat, 10,255 protein-coding genes were identified. Annotation through the Pathogen-Host Interactions (PHI) database revealed that 48% of these genes were linked to reduced virulence, 32% to unaffected pathogenicity, 9% to loss of pathogenicity, 8% to lethality, and 3% to increased virulence. This research provides valuable insights into infection-related genes, contributing to future strategies for combating common bunt disease (Gurjar et al.).

In another study, transcriptome analysis of resistant and susceptible genotypes of *Hordeum vulgare* (barley) infected with *Bipolaris sorokiniana*, the pathogen causing spot blotch, was performed to explore host-pathogen dynamics. The results showed stronger activation of MAPK signaling, plant-pathogen interactions, and plant hormone signal transduction pathways in resistant genotypes, leading to elevated expression of defense and pathogenicity-related genes. In contrast, susceptible genotypes exhibited higher expression of *B. sorokiniana* pathogenicity genes. This study sheds light on the genetic factors involved in spot blotch resistance in barley, offering new insights into host-pathogen interactions (Basak et al.). These findings will assist in developing disease mitigation strategies for crop improvement.

Biocontrol of plant pathogens is a promising alternative to antimicrobial chemicals, promoting sustainable agriculture (Jain et al., 2018). In a study on controlling *Ustilaginoidea virens*, the soil-borne fungal pathogen causing rice false smut, rice rhizospheric isolates of *Bacillus subtilis* (BR_4), *B. licheniformis* (BU_7, BU_8), and *B. vallismortis* (KU_7) exhibited antimicrobial properties. Among these, *B. vallismortis* (KU_7) was the most effective, inhibiting 44.6% of *U. virens* growth. The talc-based formulation of *B. vallismortis* (KU_7) reduced disease incidence by 20%, increased biological yield by 60.5%, and boosted grain yield by 45% (Pandey et al.). Additionally, over a 3-year study, Integrated Pest Management (IPM) practices led to a significant rise in beneficial microbes like *Trichoderma harzianum* and *Pseudomonas fluorescens*, while reducing the presence of the pathogen *Fusarium verticillioides* in rice field soil. These results underscore the effectiveness of IPM in promoting plant health and controlling diseases compared to non-IPM practices (Khokhar et al.).

## Conclusion

In conclusion, this second volume on the Climate Impact on Plant Holobiont provides a valuable addition to the earlier Research Topic. The papers in this Research Topic highlight the rapid progress in understanding the complex interactions between plants, microbes, and climate change, offering crucial knowledge for developing mitigation strategies and promoting sustainable agro-ecosystems.
